# Proliferation of Hydroelectric Dams in the Andean Amazon and Implications for Andes-Amazon Connectivity

**DOI:** 10.1371/journal.pone.0035126

**Published:** 2012-04-18

**Authors:** Matt Finer, Clinton N. Jenkins

**Affiliations:** 1 Save America's Forests, Washington D.C., United States of America; 2 Center for International Environmental Law, Washington D.C., United States of America; 3 Department of Biology, North Carolina State University, Raleigh, North Carolina, United States of America; University of Otago, New Zealand

## Abstract

Due to rising energy demands and abundant untapped potential, hydropower projects are rapidly increasing in the Neotropics. This is especially true in the wet and rugged Andean Amazon, where regional governments are prioritizing new hydroelectric dams as the centerpiece of long-term energy plans. However, the current planning for hydropower lacks adequate regional and basin-scale assessment of potential ecological impacts. This lack of strategic planning is particularly problematic given the intimate link between the Andes and Amazonian flood plain, together one of the most species rich zones on Earth. We examined the potential ecological impacts, in terms of river connectivity and forest loss, of the planned proliferation of hydroelectric dams across all Andean tributaries of the Amazon River. Considering data on the full portfolios of existing and planned dams, along with data on roads and transmission line systems, we developed a new conceptual framework to estimate the relative impacts of all planned dams. There are plans for 151 new dams greater than 2 MW over the next 20 years, more than a 300% increase. These dams would include five of the six major Andean tributaries of the Amazon. Our ecological impact analysis classified 47% of the potential new dams as high impact and just 19% as low impact. Sixty percent of the dams would cause the first major break in connectivity between protected Andean headwaters and the lowland Amazon. More than 80% would drive deforestation due to new roads, transmission lines, or inundation. We conclude with a discussion of three major policy implications of these findings. 1) There is a critical need for further strategic regional and basin scale evaluation of dams. 2) There is an urgent need for a strategic plan to maintain Andes-Amazon connectivity. 3) Reconsideration of hydropower as a low-impact energy source in the Neotropics.

## Introduction

A diminishing fraction of the world's rivers remains unaffected by humans, with dams being a leading cause of disruption [1], [2]. Nearly two-thirds of the world's large rivers are now fragmented by dams [3], leaving few major free-flowing river systems. With a heavy concentration of dams in the northern third of the world [1], [4], the Neotropics are now a primary frontier for new dam construction [5]–[9].

Hydropower offers a reliable source of domestically produced electricity to Neotropical countries, along with the chance to diversify away from thermoelectric facilities and the use of fossil fuels. However, dams may also lead to significant ecological and social impacts, both downstream and upstream of the dam site [10], [11]. In 2000, the World Commission on Dams stressed the importance of strategic assessments to minimize such environmental and social impacts of new dams [11]. Such comprehensive assessments are rare for tropical regions [5], [7], [8], and they are often constrained by the limited availability of information on potential projects and their locations [12].

We developed a strategic ecological impact assessment of planned hydroelectric dams across all six major Andean tributaries of the Amazon River (Caqueta, Madeira, Napo, Marañon, Putumayo, and Ucayali). The geographic scope spans five countries – Bolivia, Brazil, Colombia, Ecuador, and Peru – enabling analyses across entire basins and across country boundaries. Two recent global-scale analyses of rivers and water resources indicated that Amazonia shows only low to moderate levels of threat [2], [3], but those studies considered only existing dams. Our study is the first to consider the possible ecological impacts, in terms of both connectivity and forest loss, of the full governmental portfolios of proposed projects across all rivers connecting the Andes to the Amazon. We evaluated precise location data for all planned hydroelectric dams greater than 2 MW capacity to estimate regional impacts in relation to existing dams, roads, transmission lines, protected areas, and titled indigenous territories.

The current lack of strategic planning has become an important management issue in the Andean Amazon. The Amazon River has been intimately linked to the Andes mountains for over 10 million years, and major breaks in connectivity could bring severe and unpredictable impacts [13]. The Andes supply the vast majority of the sediment, nutrients, and organic matter to the main-stem Amazon, fueling a floodplain ecosystem that is among the most productive on Earth [13]–[18]. Many economically and ecologically important Amazonian fish species spawn only in Andean-fed rivers, including a number that migrate from the lowlands to the foothills [5], [13], [14], [19], [20]. Long-distance migrants include numerous large catfish (e.g., *Brachyplatystoma rousseauxii* and *Pseudoplatystoma fasciatum*) and Characins such as *Prochilodus nigricans* [13]. The Andean Amazon is also home to some of the most species rich forests and rivers on Earth [21]. The region is documented to contain extraordinary richness for the most well-studied taxa—namely amphibians, birds, mammals, and vascular plants [22]—and high levels of endemism for the understudied fishes [19]. Therefore, any dam-driven forest loss or river impacts are of critical concern.

High annual precipitation coupled with rugged topography creates significant potential for hydroelectricity across the Andean Amazon [13], [23]. The governments of Ecuador, Peru, and Bolivia are, according to official planning reports, each emphasizing hydropower as the centerpiece of medium and long-term plans to meet future energy demand. The projected new domestic demand, over 7,000 additional MW by 2020 across the three countries, stems from increasing national energy use along with efforts to replace thermoelectric facilities. Regional energy factors are important as well. Brazil is looking to meet rapidly rising energy demands over the next 20 years, but the relatively flat Brazilian Amazon is less favorable for hydropower since this form of energy production requires an elevation gradient. Therefore, projects in Brazil tend to require large, shallow reservoirs that are prone to siltation and flood vast areas [24]. Peru has signed a bilateral agreement to supply at least 6,000 MW of hydroelectric energy from Amazonian dams to Brazil over the next 30 years [20]. Bolivia is also planning several new dams by 2020 for the primary purpose of exporting energy to neighboring countries.

We collected data on dam locality, status, and size directly from government agencies and strategic planning reports (see Materials and Methods). Projects were divided into two status categories, existing and planned, along with an indication of the more advanced planned projects already under some type of contractual process. We divided project size into three categories of energy capacity: medium (2–99 MW), large (100–999 MW), and mega (≥1,000 MW).

To estimate the ecological impact of planned dams, we developed a multi-factor framework focusing on river connectivity and forest loss caused by dam-related infrastructure. This framework identified dams that would, 1) represent a major new source of river fragmentation in relation to existing dams, 2) disrupt the connectivity of free-flowing rivers that link protected Andean headwaters to the lowland Amazon, 3) require new road or 4) transmission line routes, or 5) directly cause significant environmental impacts (located within a national protected area or a confirmed long-distance migratory fish route, or flood at least 100 km^2^ of forest). For roads and transmission lines, we used a distance criterion to identify only those projects that require major new systems, not minor infrastructure additions. We defined dams that were positive for at least three factors as high impact, two factors as moderate impact, and zero or one factor as low impact. See the Materials and Methods for full details.

This framework draws from a number of important findings regarding the ecological impacts of dams and associated infrastructure. River fragmentation and subsequent loss of connectivity is one of the primary impacts [5], [6], [11], [12],[25]–[28]. Therefore, the placement of dams within a river network and relative to one another is just as important to consider as the total number and size of the dams [7], [8]. Dams may also cause forest inundation that can then lead to associated greenhouse gas emissions [11], [29]–[31], especially those in lower elevations that will need large reservoirs. The construction of access roads and transmission lines for new dams can also lead to forest loss, particularly in remote regions where extensive new systems are required. New access routes such as these, particularly roads, are well documented drivers of tropical deforestation [32].

## Results and Discussion

**Figure 1:**
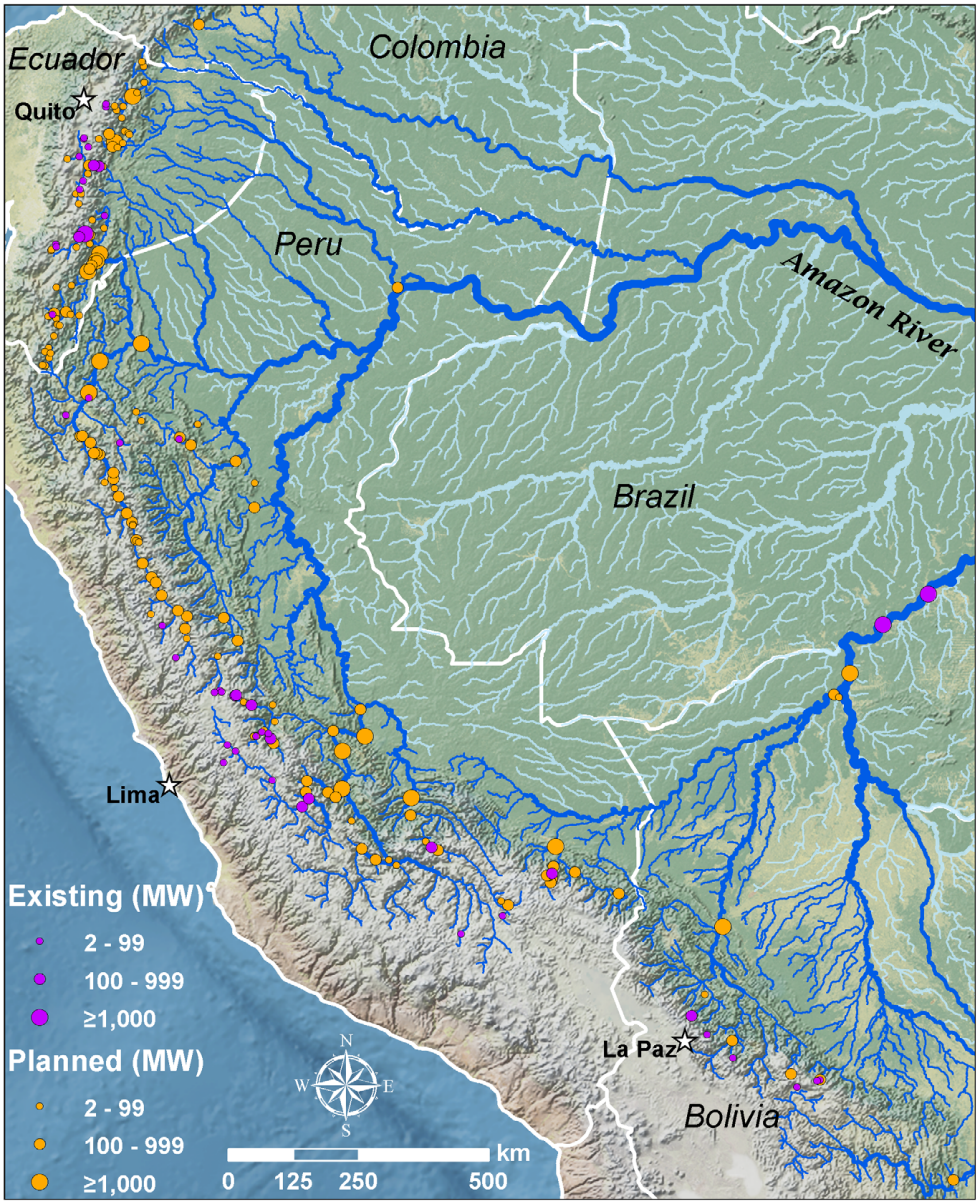
Hydroelectric dams of the Andean Amazon. Dams sorted by status (existing and planned) and size (2–99 MW, 100–999 MW, and ≥1,000 MW capacity).

There are currently 48 dams greater than 2 MW capacity in the Andean Amazon, but plans for an additional 151 such dams over the next 20 years (Table 1; Figure 1; see Figure S1 for enlarged map with labels for dams). Nearly 40% (59) of the planned dams are in advanced planning stages. Fifty-three percent (80) would be 100 MW or greater, a potential six and a half-fold increase in the number of large dams. Currently there is only one mega dam in the Andean Amazon (in Ecuador), but plans exist for 17 more. Our analysis did not include dams smaller than 2 MW capacity, largely due to a lack of consistent and comprehensive data for such small dams. Our records indicate that there are 85 such dams existing and 22 planned, mostly in Ecuador and Peru.

**Table 1:** Summary of existing and planned dams in the Andean Amazon by country and river basin.

			Capacity			
Country	Exist	Plan	(MW)	Basin	Exist	Plan
Peru	0	10	≥1000	Marañon	1	6
	7	43	100–999		3	33
	19	26	2–99		19	42
	**26**	**79**	**Total**		**23**	**81**
Ecuador	1	5		Ucayali	0	4
	3	13			6	15
	12	42			10	11
	**16**	**60**			**16**	**30**
Bolivia	0	2		Napo	0	2
	1	6			0	4
	5	2			2	13
	**6**	**10**			**2**	**19**
Colombia	0	0		Madeira	0	3
	0	1		tributaries	2	11
	0	1			5	5
	**0**	**2**			**7**	**19**
**All countries**	1	17		Caqueta	0	0
	11	63			0	1
	36	71			0	0
	**48**	**151**			**0**	**1**
				Putumayo	**0**	**0**

Existing and planned hydropower projects are concentrated in areas of high topographic relief (Figure 1). The vast majority of planned dams (84%) are above 500 m, the general start of the Andean foothills. However, the 21 dams planned below 400 m (see Figure S2) are those most likely to create large flooding reservoirs and affect long-distance migratory fish. An additional 45 dams between 400 and 1000 m (Figure S2) may impact long-distance migrants [20], [23], but we excluded this as a factor in the ecological analysis due to lack of definitive data.

### Country results

Among the four countries of the Andean Amazon, Peru has the most existing (26) and planned (79) dams over 2 MW capacity (Table 1; Figure S3). While two-thirds of the existing dams are less than 15 MW, there is a clear shift upwards as two-thirds of the planned dams are large or mega projects (Table S1). The largest existing dam in Peru, by far, is 798 MW, while 11 planned projects exceed this capacity. Further illustrating Peru's intense interest in hydroelectric energy, nearly half of all the planned dams are already in advanced planning stages.

Ecuador has the second highest totals of existing (16) and planned (60) dams (Table 1; Figure S4). Only four existing dams are larger than 100 MW, including the only current dam in the Andean Amazon that exceeds 1,000 MW, while there are 18 planned large dams, including five more mega dams (Table S1). Thirty percent of the planned dams are in advanced planning.

Bolivia has fewer existing (6) and planned (10) dams (Table 1; Figure S5), although several of the existing ones are complexes with multiple dams. While only one existing dam exceeds 100 MW, eight of the planned dams are large or mega projects.

The Colombian Amazon has no existing dams, and just one large dam has been proposed (Figure S4). We were only able to find definitive information for one additional planned medium dam, but a recent report indicates there may be more [33]. This lack of existing and advanced planned dams matches a previous finding in regards to the hydrocarbon sector that Colombia represents the most pristine section of the Andean Amazon from the perspective of energy development [34].

### River basin results

**Figure 2:**
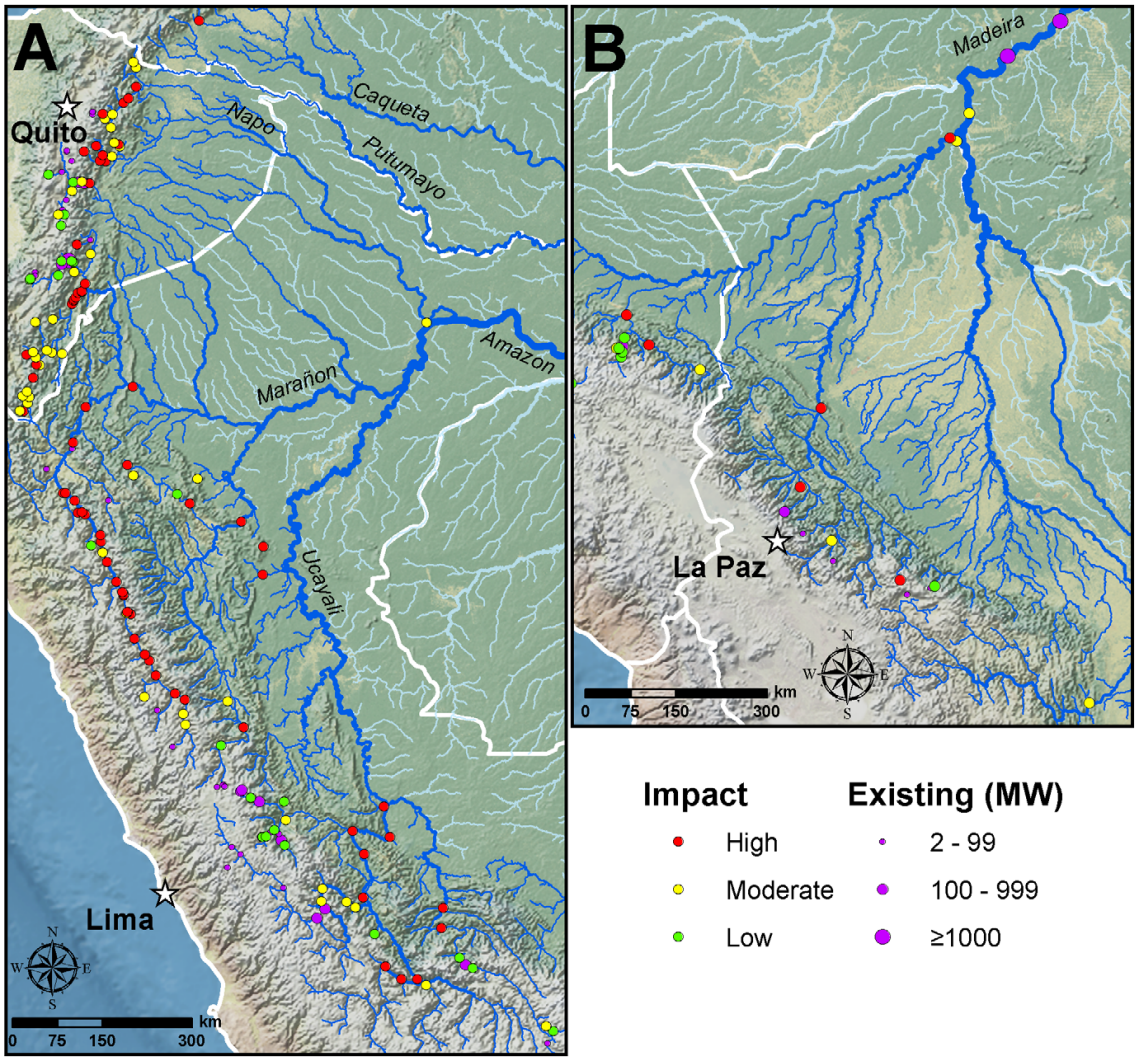
Results of ecological analysis. (A) Results for tributaries originating in the Colombian, Ecuadorian, and northern Peruvian Andes. (B) Results for tributaries originating in the Bolivian and southern Peruvian Andes.

Considering the six major Andean tributaries of the Amazon (Caqueta, Madeira, Napo, Marañon, Putumayo, and Ucayali), new dams threaten to break the now largely free-flowing nature of five. Most threatened are rivers originating in the Ecuadorian and northern Peruvian Andes, while those in Colombia are the least threatened (Table 1; Figure 2A).

More than half (81) of all planned dams are located on the Marañon River and its sprawling tributaries (including the Huallaga, Pastaza, and Zamora Rivers) across Ecuador and Peru (Table 1; Figure S6). Much of the existing hydropower for Ecuador comes from four large dams on two northern tributaries of the Marañon, but the rest of the river complex is free-flowing. However, there are plans for over 60 new dams on these free-flowing stretches. In April 2011, the outgoing administration of President Alan Garcia issued a decree declaring that the construction of 20 of these dams, all located on the main-stem, were in the national interest. All 20 of these prioritized Marañon dams would exceed 100 MW, including three new mega dams (Escuprebraga, Rentema, and Manseriche). Also noteworthy is a cluster of large and mega dams slated for the Zamora River and the first large dams for the Huallaga.

The Ucayali River complex in Peru has the second highest number of planned dams with 30 (Table 1; Figure S7). Six large dams on the upper tributaries of the Ucayali now provide much of Peru's hydropower. There are plans for an additional 19 large dams, including four mega dams near the confluence of the two major rivers forming the main-stem Ucayali (Tambo and Urubamba Rivers). Four of these dams (Mainique, Paquitzapango, Tambo 40, and Tambo 60) are most often discussed in terms of projects being offered under the Peru-Brazil energy agreement.

The Napo River complex is almost entirely free-flowing. The only two existing dams are less than 10 MW and do no not occur on major waterways (Figure S8). However, 19 additional dams are planned, including four large and two mega dams (Table 1). This includes the largest mega dam slated for the Ecuadorian Amazon, Coca Codo Sinclair. Only one of the Napo dams, Mazan, is outside of Ecuador. This dam is located near Iquitos, Peru, and is noteworthy in that initial designs do not call for the damming of the Napo River. It will instead just divert a portion of the water for energy production [35].

In Colombia, the Putumayo River is the only major Andean-born Amazon tributary with no existing or planned dams >2 MW capacity. The Caqueta River also has no existing dams, but one large planned dam (Andaquí). However, the Colombian Environment Ministry denied the environmental license for Andaquí in 2009.

Connectivity between the Amazon and the Bolivian and southern Peruvian Andes is being broken with the construction of two mega dams in Brazil on the upper Madeira River (Santo Antônio and Jirau) (Figure 2B, Figure S9). Fourteen more large and mega dams are planned for tributaries of the Madeira. One of these, the controversial Inambari dam of Peru, is, along with the four dams noted above, one of the projects often discussed under the Peru-Brazil energy agreement. The three largest planned dams in Bolivia (Rio Madera, Angosto del Bala, and Cashuela Esperanza) are also designed for energy export. The string of four dams on the Brazil-Bolivia border—Santo Antônio, Jirau, Rio Madera, and Cashuela Esperanza—are the only major hydroelectric dam projects in the study area directly associated with the IIRSA Initiative (an institutional mechanism for the coordination of intergovernmental actions by South American countries in regards to transportation, energy and communications projects).

### Impact Analysis

The ecological impact analysis classified 71 (47%) of the planned dams as high impact, 51 (34%) as moderate impact, and 29 (19%) as low impact (Figure 2; Table 2). Considering the individual factors (Table S1), 82% of new dams would represent a high or moderate fragmentation event, while 60% would cause the first major break in connectivity between protected Andean headwaters and the lowland Amazon. Deforestation would be a major issue for many dams, with 36% requiring new roads and 79% needing new transmission line routes (Table S1). Eleven dams would directly impact a protected area.

**Table 2:** Summary of estimated ecological impact and potential energy capacity from low and moderate impact dams in relation to projected 2020 demand.

	Ecological Impact (No. of dams)			Low Impact (MW)	Mod Impact (MW)	New Demand by 2020 (MW)	2020 Demand met by low/mod
	Low	Mod	High				
Peru	18	19	42	1473	3565	3526	143%
Ecuador	10	26	24	1074	1015	3200	65%
Bolivia	1	5	4	127	3662	650	583%
Colombia	0	1	1	0	2	-	-
TOTAL	29	51	71	2674	8244	7376	148%

Examples of high impact dams include: Andaquí in Colombia, which would represent the first major break in connectivity of the Caqueta River and flood a National Park; Coca Codo Sinclair in Ecuador, which would be the first major disruption of downstream sediment flow for a major tributary of the Napo River (upstream fish migration naturally blocked at this same point by San Rafael Falls) and require extensive road-building and transmission line construction in primary forest; all five dams in Peru associated with the Peru-Brazil energy agreement (Inambari, Mainique, Paquitzapango, Tambo 40, and Tambo 60) due to flooding, fragmentation, and required infrastructure; many of the large dams slated for the Marañon River, most notably Manseriche for the first major break in connectivity of the main-stem and impacts on migratory fish; Angosto del Bala and Cashuela Esperanza in Bolivia for extensive flooding, including major impacts on Madidi National Park in the case of the former. Low impact dams are primarily those that take advantage of existing infrastructure to minimize river fragmentation and road construction, such as Sopladora and Cardenillo in Ecuador and Curibamba and San Gaban III in Peru.

Some of the most controversial projects involve direct impacts on indigenous communities. Communities upstream of new dams face flooding and displacement issues, while downstream communities may be impacted by the disruption of the river's natural flow [36]. As a first step in assessing these social impacts, we evaluated dams in relation to officially titled indigenous territories. Forty dams (26%) would be constructed immediately upstream or downstream of a titled indigenous territory (Table S1). Interestingly, the ecological impact analysis identified all but one of these dams as high or moderate impact. We did not formally include this factor into the ecological framework as this issue already has a long history of building towards a social framework. The International Labour Organization's Indigenous and Tribal Peoples Convention 169 (ILO 169) of 1989, an international instrument ratified by all four Andean Amazon countries, mandates consultation with impacted communities with the aim of achieving consent [37]. Peru's 2011 consultation law is explicitly based on ILO 169 [38] and Ecuador's 2008 Constitution stipulates free, prior and informed consultation [39]. Additionally, the 2007 United Nations Declaration on the Rights of Indigenous Peoples states that consultation with impacted communities be conducted in order to obtain their free and informed consent prior to the approval of any development project affecting water resources [40].

### Future work

Future work could build upon our analysis by incorporating data on additional important ecological and social factors. More data on reservoir size and associated flooding could enhance precision of the impact analysis. Due to lack of available information, we were only able to account for the largest reservoirs that would require flooding of at least 100 km^2^ of forest. Future work is also needed to incorporate better data on long-distance migratory fish routes, as we used a conservative measure of areas documented to affect migration.

An in-depth analysis on the location of new dams in relation to specific ecosystems could help elucidate possible additional ecological impacts. We conducted an initial analysis using the global ecoregion dataset [41] and NatureServe-defined Ecological Systems [42], [43]. The Ecoregions are a broad global-scale classification system while Ecological Systems represent a more refined system for the Andean Amazon region. We found that existing and planned dams occur in 14 Ecoregions and 30 Ecological Systems, respectively (Table S2), but more extensive work is needed to precisely determine relative impacts.

It is becoming increasingly urgent to understand better the impacts of dams in the rugged Andes, a global center of endemism [44]. Rugged sites of high dam suitability may also be the sites of highest probability of localized speciation. For example, a recent study estimates that nearly 40% of fish species in the Tropical Andes are endemic to the region [19]. Furthermore, many Andean dams are run-of-river projects that divert water from the main channel for a number of kilometers before returning the water further downstream. These de-watered reaches often experience significant flow reductions and, along with the stretches immediately below where the diverted water is reintroduced, become drastically different living environments [7]. As species distribution data becomes more available, Tropical Andean fishes are among the most understudied vertebrates in the world [19], it will be important to consider the location of Andean dams in relation to restricted-range species [45].

### Policy implications

These findings have three important implications for policy. First, as regional governments promote hydropower as the centerpiece of long-term energy plans, a shift towards more strategic, multi-factor planning and assessment could reduce potentially profound ecological impacts. Under the present system of project-level environmental impact assessment typical in the Neotropics, projects are evaluated mainly on an individual basis prior to construction [8]. Similarly, the new Hydropower Sustainability Assessment Protocol [46] largely focuses on individual projects [47], [48]. In contrast, we advance a framework to evaluate impacts in terms of both connectivity and forest loss at a basin and regional scale. We believe this framework could be a useful tool for governments in terms of how to incorporate data across entire basins, particularly the complicated matter of trans-boundary analysis. With active decentralization efforts in the region, much planning is being scaled down to the departmental/provincial level, making strategic trans-boundary analysis even more challenging. Our approach may allow decision makers to complement localized planning with regional data to identify more effectively the most sustainable, and the most destructive, dam locations. Otherwise, under the business-as-usual scenario, planning and construction of dams in the Andean Amazon will continue as a chaotic, project-focused endeavor with little regard for the larger regional picture.

Second, there is clearly a need for a strategic plan to maintain free-flowing connectivity from the Andean highlands to the Amazon lowlands. This would involve safeguarding remaining free-flowing major river systems from hydropower development, from headwaters to estuary, a task complicated by the complex multi-nation trajectory of Andean-born rivers. With the main-stem Madeira River now losing connectivity due to construction of two mega dams, there is increased importance and urgency to take a closer look at planned dams on the Marañon, Ucayali, Napo, and Caqueta Rivers with the aim of ensuring sufficient free-flowing stretches between the Andes and Amazon. This initiative would likely require the creation of a new multi-nation commission or task force, as we are unaware of any current entity tasked with evaluating issues across all Andes-Amazon river basins. Peru has two national entities that could potentially serve as initiators of such an initiative: the Peruvian Amazon Research Institute (http://www.iiap.org.pe) and the Interregional Amazon Council (http://www.ciam.org.pe). Both are working to collect and analyze information across departmental boundaries in Peru, so it may be a natural extension to expand across national boundaries as well.

Third, given the rarity of low impact dam sites found (just 19%), we challenge the notion of Neotropical hydropower being a widespread low impact energy source. Institutions and instruments that support Neotropical dams, such as international financial institutions and the Clean Development Mechanism (CDM), should consider the wide array of factors examined here during project evaluations. Otherwise, tropical rivers and forests may increasingly be at risk from otherwise well-intentioned strategies to mitigate climate change. For example, hydropower is currently the most common type of project vying for carbon credits through the CDM [49].

Andean nations could meet a substantial percentage of expected energy needs by prioritizing only low, and perhaps moderate, impact dams (Table 2). The non-Amazonian watersheds of these countries also possess substantial hydroelectric potential. Replicating our analysis in these zones could identify additional low impact projects to complement Amazon production.

## Materials and Methods

We collected information on existing and proposed hydroelectric dams from two sources: (1) directly from government ministries following official information requests, and (2) publicly available government reports. In each country, we submitted official data requests to the appropriate governmental ministry during 2010, and then followed-up on those requests until receipt of the data.

For each hydroelectric dam project in each country, we identified the location, project status (existing or planned), and size (in megawatts). For project status, we further distinguished the more advanced planned projects already under some type of contractual process. For project size, the maximum design capacity in megawatts was used as a consistent measure due to lack of consistent data on other potential measures, such as dam wall size, reservoir size, general type (storage vs. run-of-river), or water flow impacted. We classified dams into three categories: medium (2–99 MW), large (100–999 MW), and mega (≥1000 MW). We omitted from the analysis all dams <2 MW, largely due to a lack of consistent and comprehensive data for such small dams. We also omitted irrigation and drinking water dams due to lack of consistent data across countries.

For Ecuador, data on existing and planned hydroelectric dams were obtained from the Department of Planning of the National Council of Electricity (Dirección de Planificación del Consejo Nacional de Electricidad; CONELEC). Advanced projects were those under the following types of contractual processes: contrato, certificado, and tramite. Localities for less advanced hydroelectric dams are from the 2009 Ecuador Inventory of Energy Resources for Electric Generation [50]. Additional information for future plans was gleaned from the 2009–2020 Master Electrification Plan [51]. The projected 2020 electric energy demand was based on the “escenario medio” from the 2009–2020 Master Electrification Plan [51].

For Peru, data for both existing and planned hydroelectric dams were obtained from the Department of Electricity of the Ministry of Energy and Mines (Dirección General de Electricidad del Ministerio de Energía y Minas). Advanced projects were those under the following types of contractual processes: definitiva and temporal. Information for less advanced hydroelectric dams was obtained from the Evaluation of National Hydroelectric Potential 1973–1982 [52], the Elaboration of Executive Summaries for Hydroelectric Plants with Potential for Export to Brazil [53], and Supreme Decree N° 020-2011-EM [54]. Additional information for existing and future hydroelectric projects was gleaned from the 2008 Annual Statistical Report for Electricity [55], 2009 Electric Sector Promotion Document [56], Reference Plan for Electricity 2008–2017 [57], and Portfolio of Generation and Transmission Projects in the National Interconnected Electric System [58]. Statuses of all projects were verified as of June 1, 2011. The projected 2020 electric energy demand was based on the “Escenario de Demanda Medio” from the Reference Plan for Electricity 2008–2017 [57].

For Bolivia, data for planned hydroelectric projects are from the National Electricity Company (Empresa Nacional de Electricidad; ENDE). Additional information for planned projects is from the 2010–2015 Strategic Institutional Plan [59], Projections of the Energy Sector 2010–2015 [60], and Energy Development Plan: Analysis of Scenarios for 2008–2027 [61]. General information for existing hydroelectric dams was also obtained from the National Energetic Balance 2000–2007 [62] and 2008 Annual Report [63]. The projected 2020 electric energy demand was based on the 2007–2014 strategic electricity plan [64].

For Colombia, information on existing and planned hydroelectric projects was gleaned from the Portfolio of Energy Generation Projects [65], Statistical Bulletin of Energy of Mines and Energy 2003–2008 [66], and Referential Expansion Plan for Generation and Transmission 2009–2023 [67].

Localities of two dams under construction on the upper Madeira River in Brazil were obtained from International Rivers [68].

We verified that dam locations matched the locations of existing rivers by comparing the locality to the HydroSHEDS database of rivers [69] and to available satellite imagery in Google Earth. HydroSHEDS is currently the most detailed and comprehensive global database with consistent coverage of topographically derived data with hydrological modeling applications. In HydroSHEDS, river size is defined as the number of cells upstream from a particular location on the river.

We evaluated the potential ecological impacts of each planned dam using a five-factor analysis. The factors were: 1) fragmentation index, 2) Andes-Amazon connectivity, 3) transmission line access, 4) road access, and 5) significant known ecological issue.

The fragmentation index classified each planned dam as causing low, moderate, or high levels of fragmentation. Low fragmentation was defined as, a) a new dam in the immediate vicinity of an existing large or mega dam, or b) a medium dam in the immediate vicinity of another medium dam. Moderate fragmentation was defined as, a) a new large or mega dam that is not in the immediate vicinity of an existing large or mega dam, but that is on the same main channel of a large or mega dam, b) a new large or mega dam in the immediate vicinity of an existing medium dam, or c) a new medium dam not in the immediate vicinity of an existing dam. High fragmentation was defined as a new large or mega dam not in the immediate vicinity or on the same main channel of an existing large or mega dam. Immediate vicinity was defined as being within 25 km upstream or downstream of an existing dam, or until a major tributary joined in the downstream direction. A major tributary was any river with >2,000 upstream cells in HydroSHEDS. The fragmentation analysis is based largely on two assumptions. The first, based on expert interviews, is that the dam size classes reflect relative impacts differences: dams over 100 MW tend to introduce a new level of impact, as do dams over 1,000 MW. The second assumption, as best elaborated in the Serial Discontinuity Concept, is that rivers have an innate tendency to reset ecological conditions toward natural or unregulated conditions as distance downstream from the dam increases and/or unregulated tributaries enter the system [70].

The remaining four factors were yes or no responses. For connectivity, a yes was defined as a planned dam that would represent the first major disruption of hydrological connectivity between protected Andean headwaters and the lowland Amazon. Protected headwaters are those within a protected area recognized by the national government. The Santo Antônio and Jirau dams, currently under construction on the upper Madeira River in Brazil, were considered as existing in the connectivity analysis. For transmission line and road access, a yes was defined as a planned dam more than 3 km from an existing road or transmission line, thus requiring major new road or transmission line installation. For the known ecological issue factor, a yes was defined as a planned dam that is within or that would directly impact a protected area recognized by the national government, affect long-distance migratory fish, or that would require flooding of at least 100 km^2^ of forest.

For the fragmentation and connectivity analysis, we considered each planned dam only in relation to existing dams. As new dams are constructed, the analyses will need updating as results could change.

Roads data are from the Ministerio de Transporte y Obras Públicas in Ecuador, Ministerio de Transportes y Communicaciones in Peru, Instituto Geografico Militar in Bolivia, and the Instituto Nacional de Vías in Colombia. Transmission line data are from the Consejo Nacional de Electricidad in Ecuador, the Ministerio de Energía y Minas in Peru, and the Comité Nacional de Despacho de Carga in Bolivia. Protected areas data are from the Ministerio de Ambiente in Ecuador, Servicio Nacional de Áreas Naturales Protegidas por el Estado in Peru, Ministerio de Medio Ambiente y Aguas in Bolivia, and the World Database on Protected Areas for Colombia.

For the final score, we classified a dam with three or more high/yes marks as high impact, two or more high/moderate/yes marks as moderate impact, and all others as low impact.

To assess potential impacts on indigenous peoples, we overlaid planned dams on maps of titled indigenous lands published by the Red Amazónica de Información Socioambiental Georreferenciada [71]. Dams were categorized as yes if their planned location was within 5 km upstream or downstream of a titled indigenous community or territory.

The background layer for the figures uses Natural Earth data (www.naturalearthdata.com).

## Supporting Information

Figure S1 **Enlarged high-resolution version of** **Figure 1** **, including labels of all dams included in the analysis.** The reader will need to zoom into the map to see specific dam information. Labels for dams correspond to those in Table S1.
(PDF)

Figure S2 **General elevation category for all planned and existing dams considered in the study.**
(TIF)

Figure S3 **Hydroelectric dams of the Peruvian Amazon.** Dams are grouped by status (Existing, Planned, and Advanced Planned) and size (2–99 MW, 100–999 MW, and ≥1,000 MW capacity). Advanced Planned corresponds to projects already under some type of contractual process.
(TIF)

Figure S4 **Hydroelectric dams of the Ecuadorian and Colombian Amazon.** Dams are grouped by status (Existing, Planned, and Advanced Planned) and size (2–99 MW, 100–999 MW, and ≥1,000 MW capacity). Advanced Planned corresponds to projects already under some type of contractual process.
(TIF)

Figure S5 **Hydroelectric dams of the Bolivian Amazon.** Dams are grouped by status (Existing, Planned, and Advanced Planned) and size (2–99 MW, 100–999 MW, and ≥1,000 MW capacity). Advanced Planned corresponds to projects already under some type of contractual process.
(TIF)

Figure S6 **Hydroelectric dams of the Marañon River Basin.** Dams are grouped by status (Existing, Planned, and Advanced Planned) and size (2–99 MW, 100–999 MW, and ≥1,000 MW capacity). Advanced Planned corresponds to projects already under some type of contractual process.
(TIF)

Figure S7 **Hydroelectric dams of the Ucayali River Basin.** Dams are grouped by status (Existing, Planned, and Advanced Planned) and size (2–99 MW, 100–999 MW, and ≥1,000 MW capacity). Advanced Planned corresponds to projects already under some type of contractual process.
(TIF)

Figure S8 **Hydroelectric dams of the Napo River Basin.** Dams are grouped by status (Existing, Planned, and Advanced Planned) and size (2–99 MW, 100–999 MW, and ≥1,000 MW capacity). Advanced Planned corresponds to projects already under some type of contractual process.
(TIF)

Figure S9 **Hydroelectric dams of the Andean tributaries of the Madeira River Basin.** Dams are grouped by status (Existing, Planned, and Advanced Planned) and size (2–99 MW, 100–999 MW, and ≥1,000 MW capacity). Advanced Planned corresponds to projects already under some type of contractual process.
(TIF)

Table S1 **List of all planned dams considered in the study and their key information and ecological impact scores.**
(DOC)

Table S2 **Ecological Systems and Ecoregions of all planned dams considered in the study.**
(XLS)
