# Supplementary figures and images for: Proliferation of Hydroelectric Dams in the Andean Amazon and Implications for Andes-Amazon Connectivity
Source: PLoS One. 2012 Apr 18;7(4):e35126. doi: 10.1371/journal.pone.0035126 (PMC3329437; doi:10.1371/journal.pone.0035126)

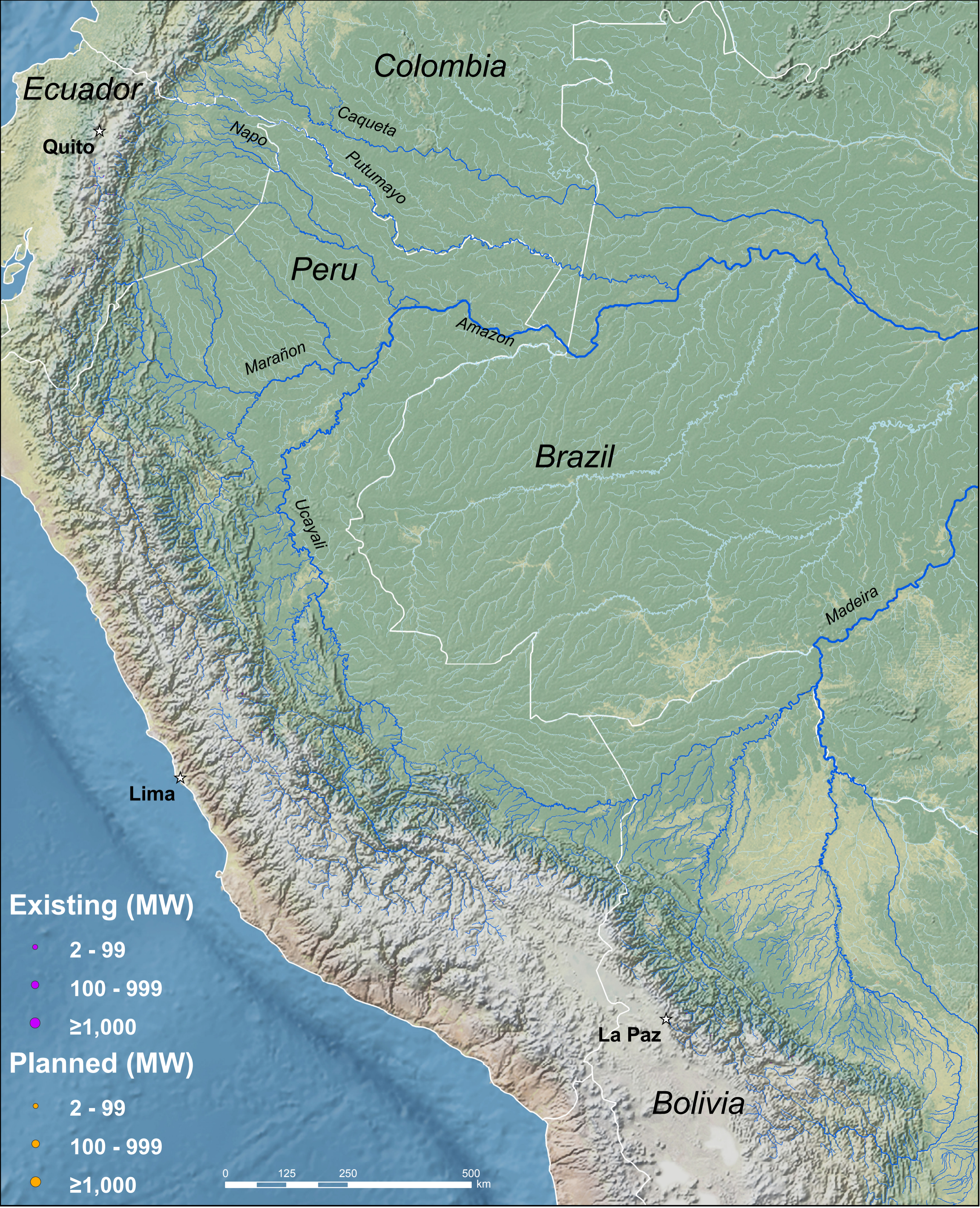

Supplement: Figure S1 — Enlarged high-resolution version of Figure 1 , including labels of all dams included in the analysis. The reader will need to zoom into the map to see specific dam information. Labels for dams correspond to those in Table S1. (PDF) [file pone.0035126.s001.pdf]

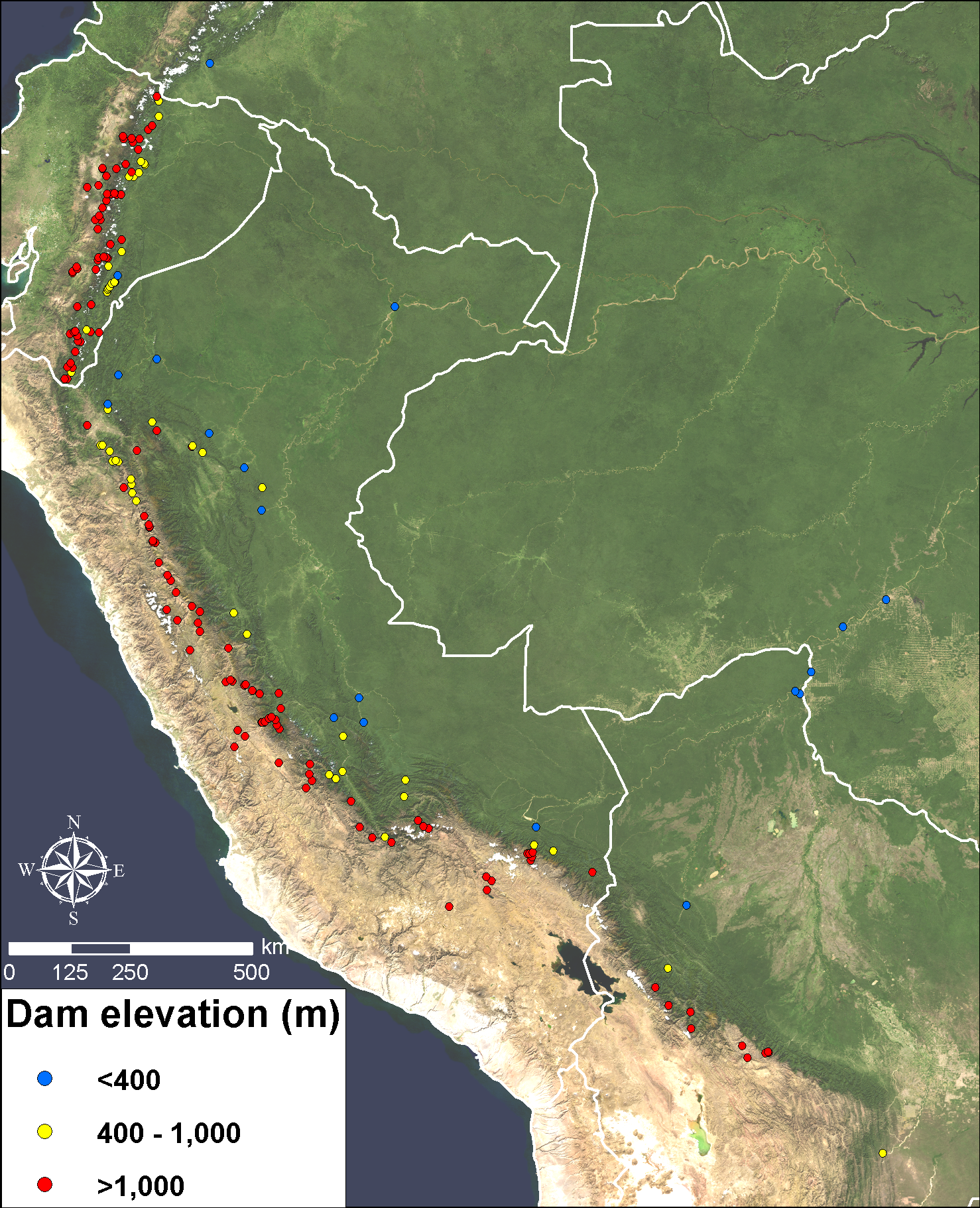

Supplement: Figure S2 — General elevation category for all planned and existing dams considered in the study. (TIF) [file pone.0035126.s002.tif]

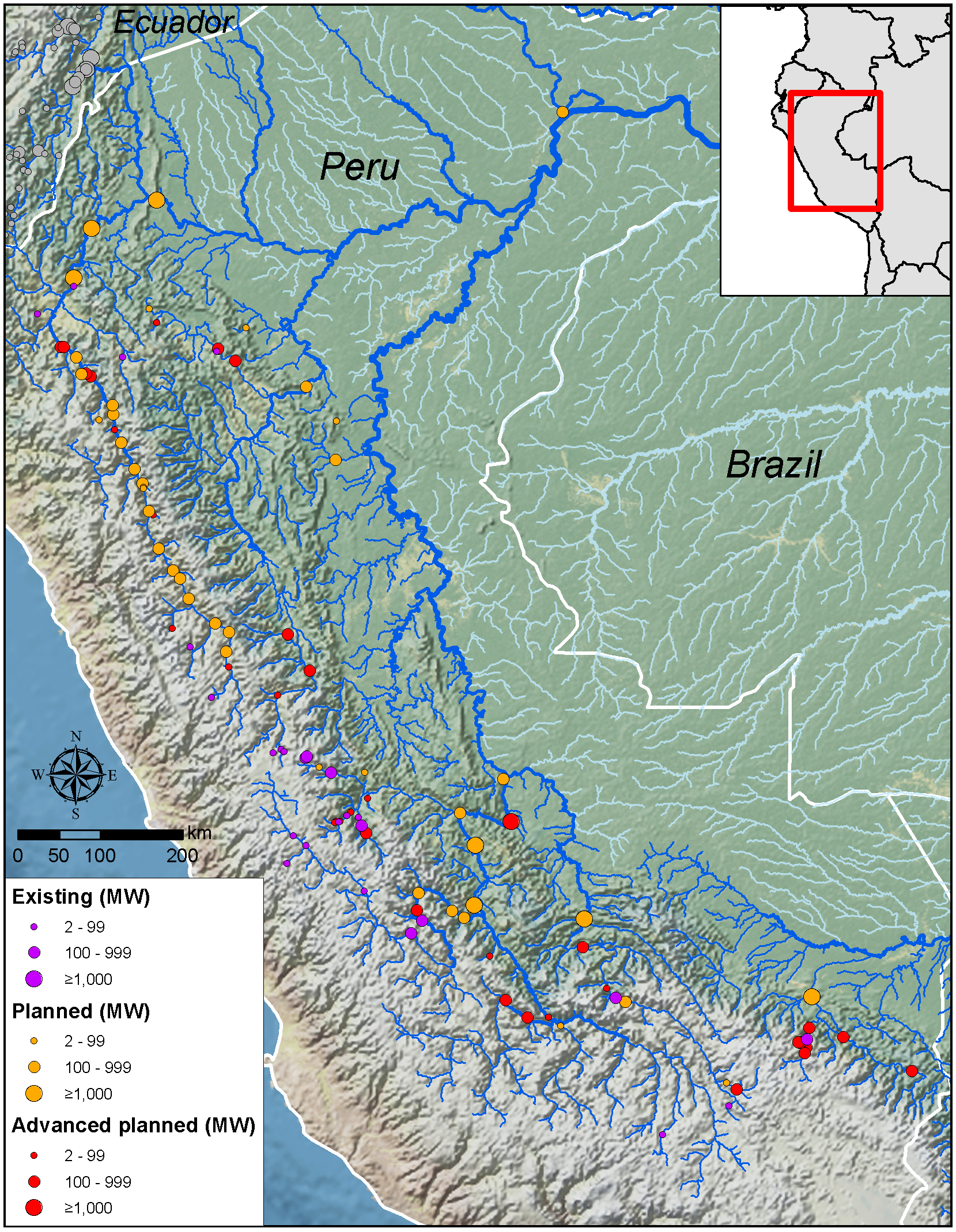

Supplement: Figure S3 — Hydroelectric dams of the Peruvian Amazon. Dams are grouped by status (Existing, Planned, and Advanced Planned) and size (2–99 MW, 100–999 MW, and ≥1,000 MW capacity). Advanced Planned corresponds to projects already under some type of contractual process. (TIF) [file pone.0035126.s003.tif]

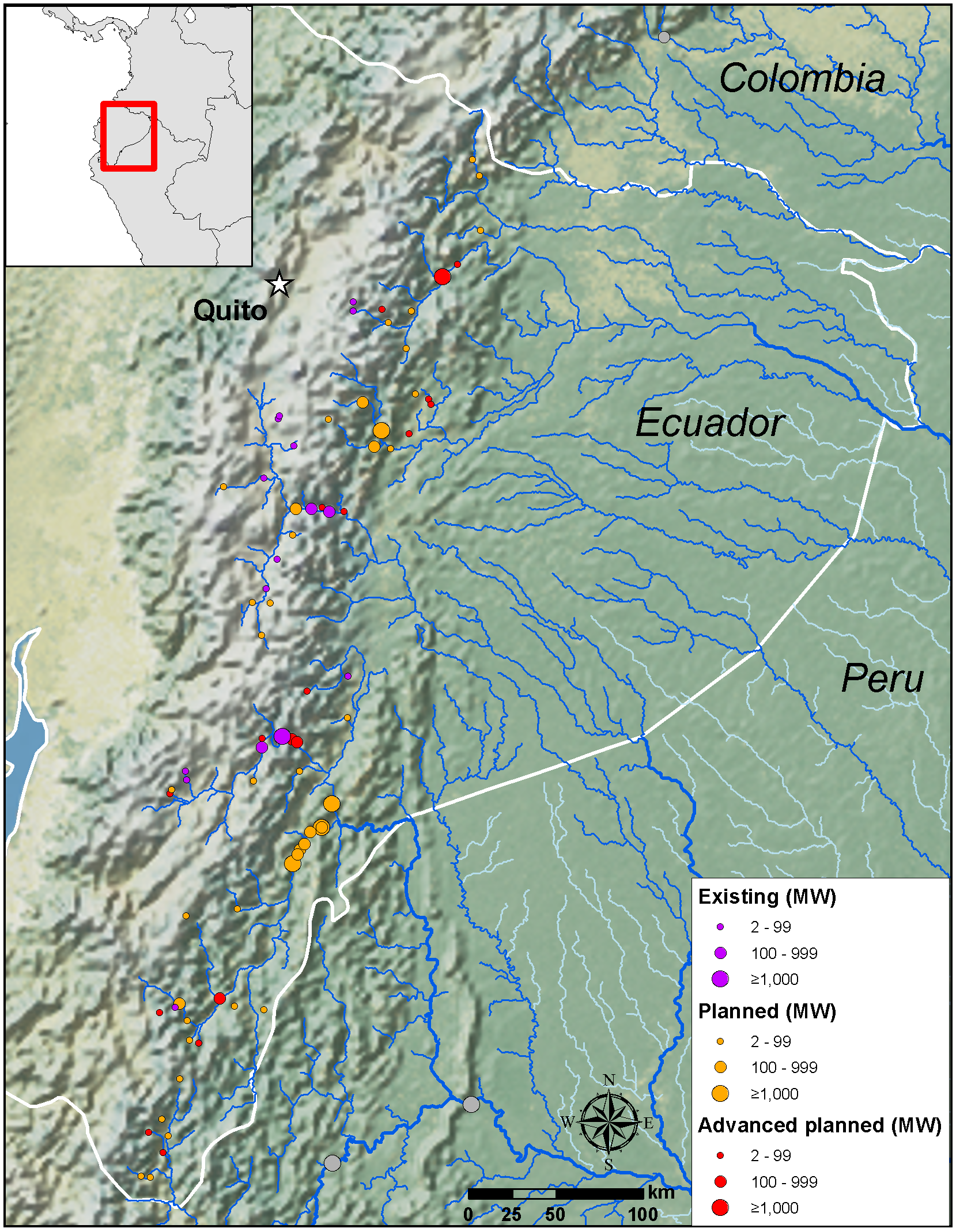

Supplement: Figure S4 — Hydroelectric dams of the Ecuadorian and Colombian Amazon. Dams are grouped by status (Existing, Planned, and Advanced Planned) and size (2–99 MW, 100–999 MW, and ≥1,000 MW capacity). Advanced Planned corresponds to projects already under some type of contractual process. (TIF) [file pone.0035126.s004.tif]

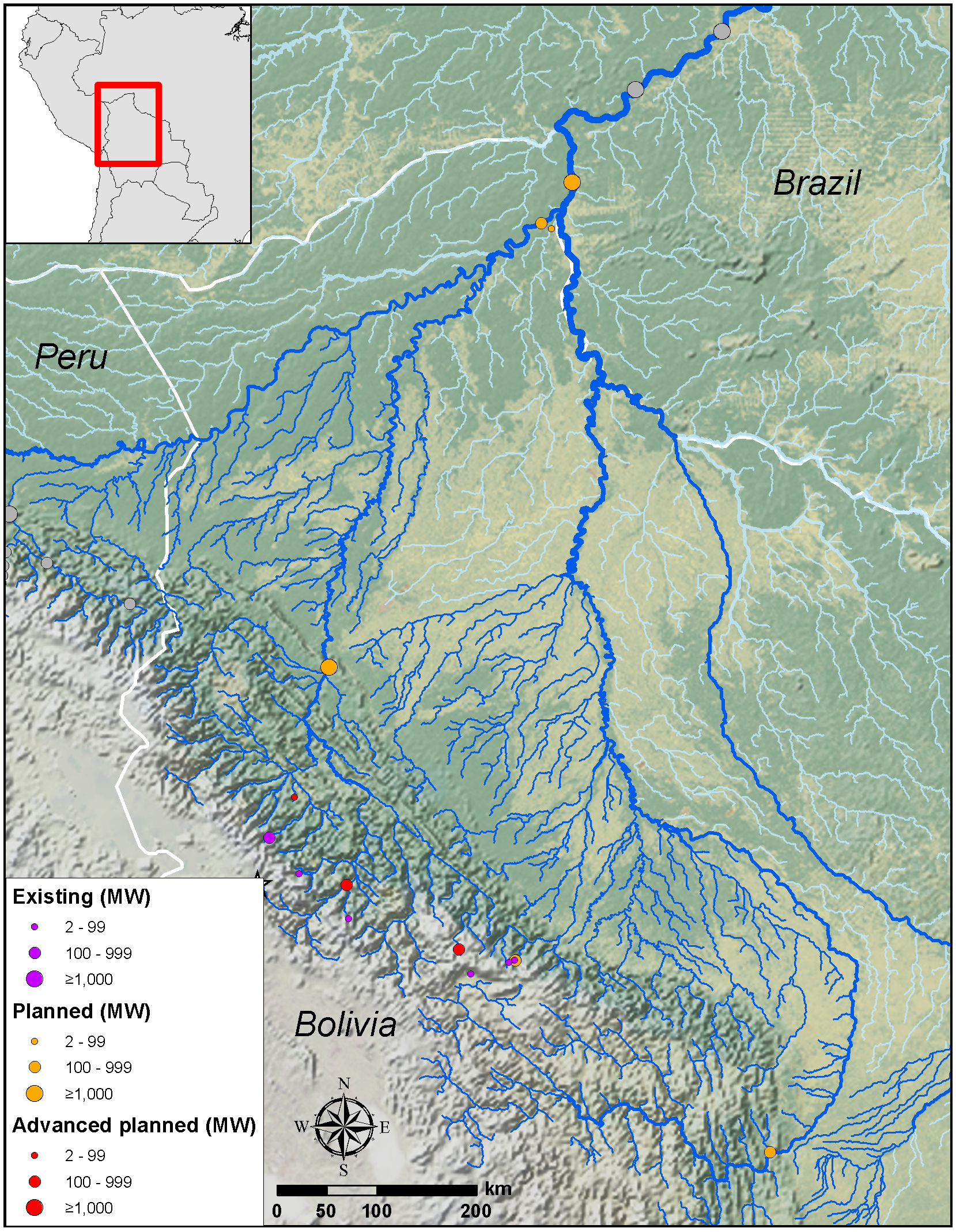

Supplement: Figure S5 — Hydroelectric dams of the Bolivian Amazon. Dams are grouped by status (Existing, Planned, and Advanced Planned) and size (2–99 MW, 100–999 MW, and ≥1,000 MW capacity). Advanced Planned corresponds to projects already under some type of contractual process. (TIF) [file pone.0035126.s005.tif]

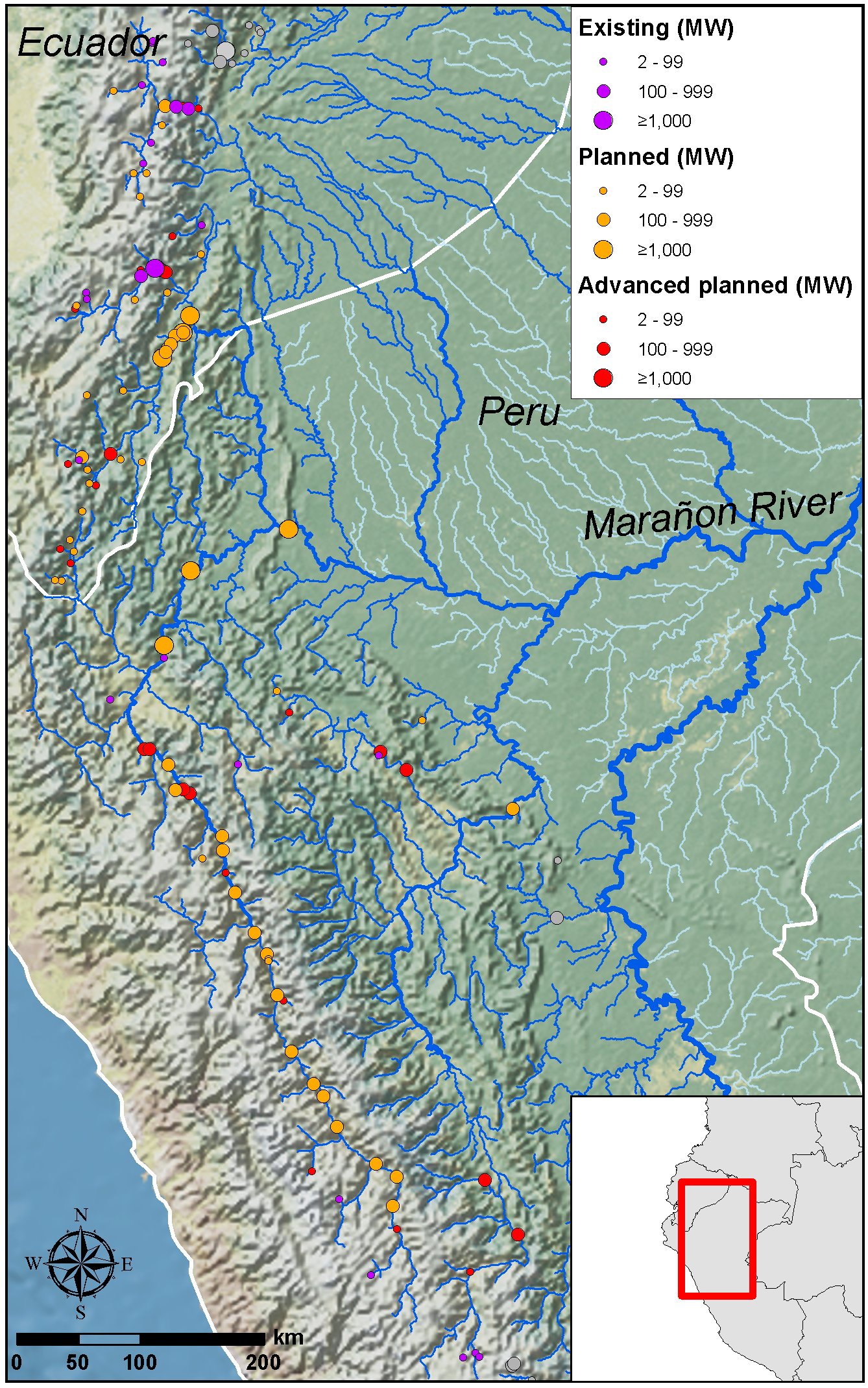

Supplement: Figure S6 — Hydroelectric dams of the Marañon River Basin. Dams are grouped by status (Existing, Planned, and Advanced Planned) and size (2–99 MW, 100–999 MW, and ≥1,000 MW capacity). Advanced Planned corresponds to projects already under some type of contractual process. (TIF) [file pone.0035126.s006.tif]

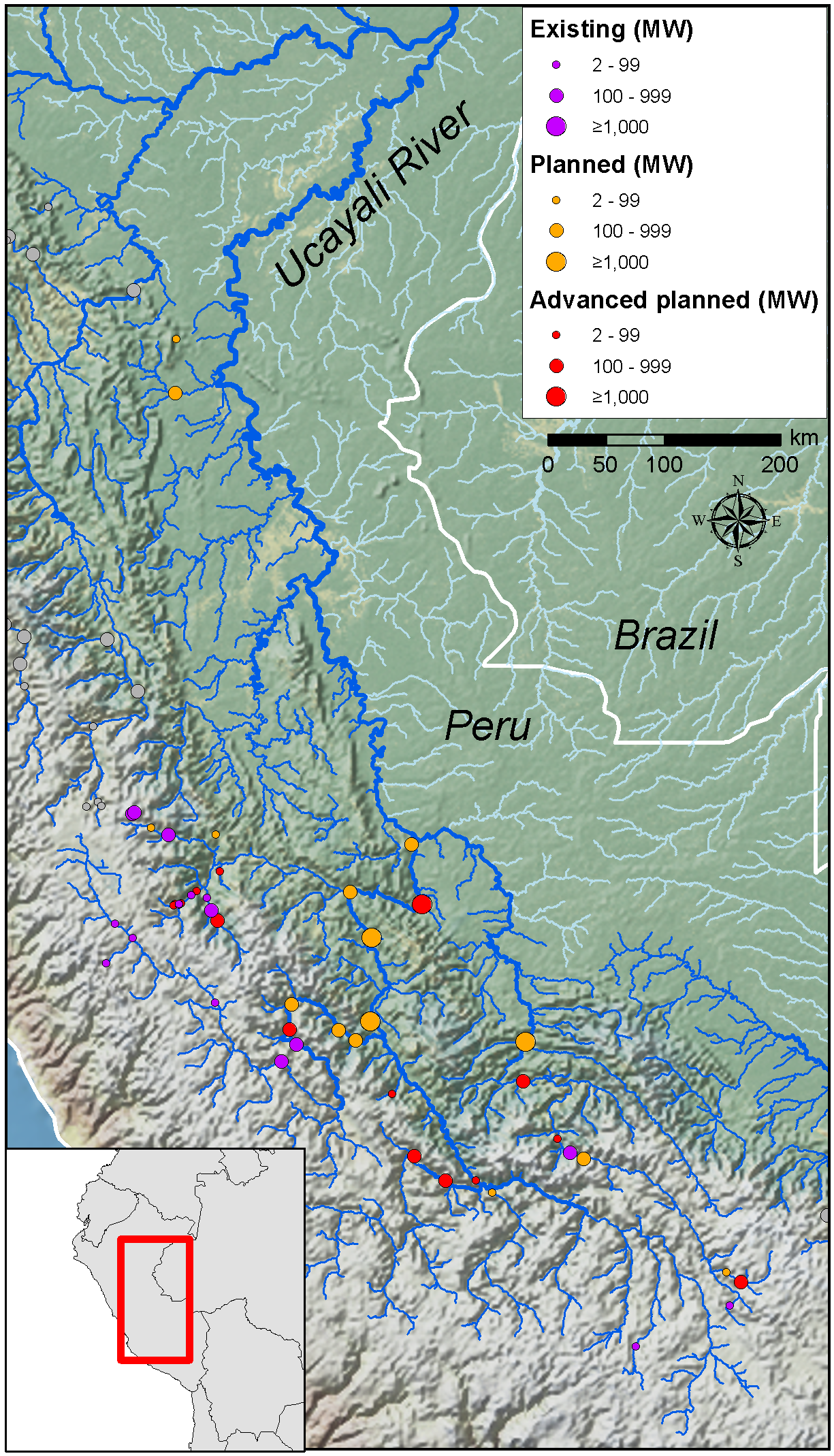

Supplement: Figure S7 — Hydroelectric dams of the Ucayali River Basin. Dams are grouped by status (Existing, Planned, and Advanced Planned) and size (2–99 MW, 100–999 MW, and ≥1,000 MW capacity). Advanced Planned corresponds to projects already under some type of contractual process. (TIF) [file pone.0035126.s007.tif]

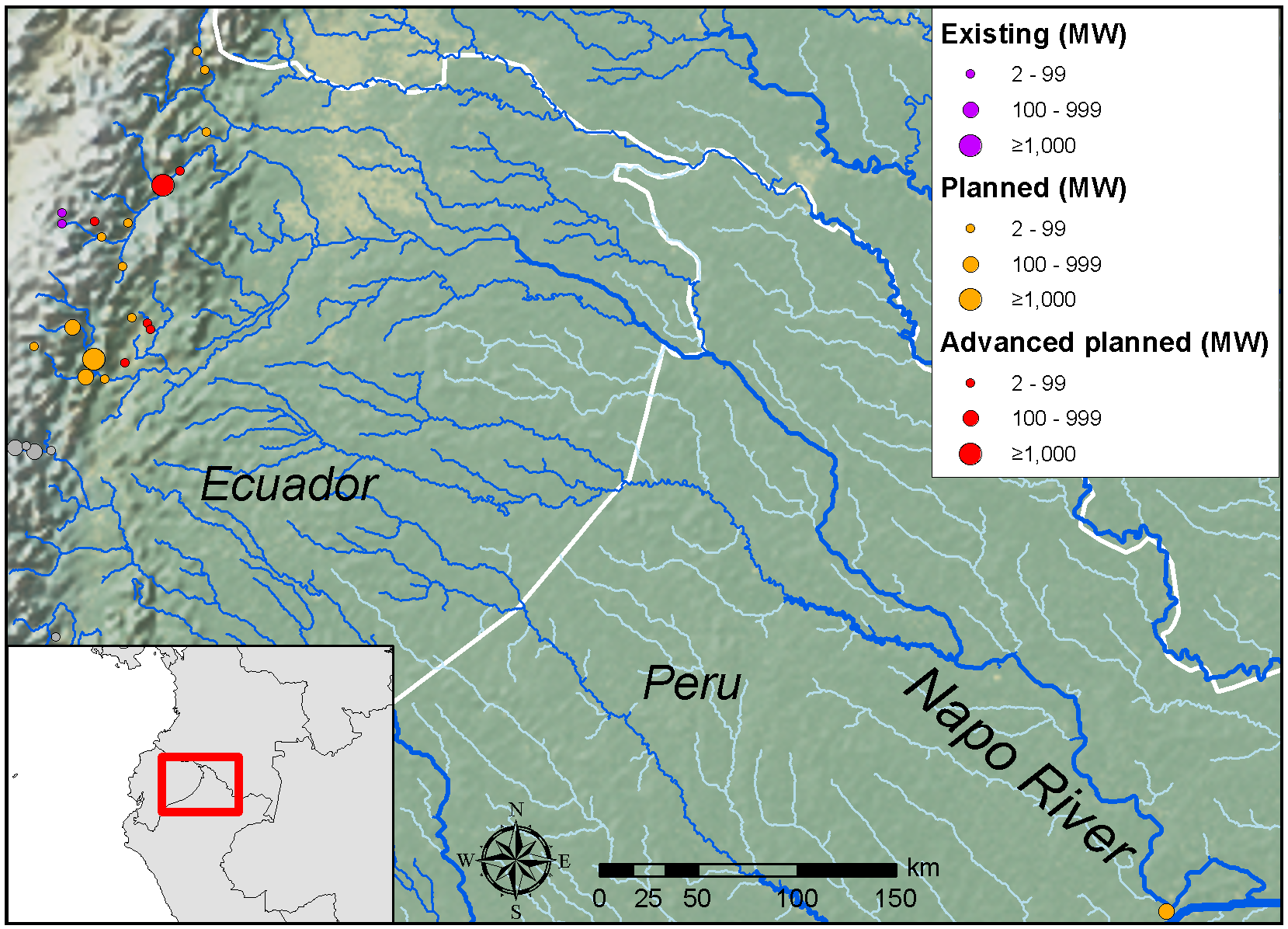

Supplement: Figure S8 — Hydroelectric dams of the Napo River Basin. Dams are grouped by status (Existing, Planned, and Advanced Planned) and size (2–99 MW, 100–999 MW, and ≥1,000 MW capacity). Advanced Planned corresponds to projects already under some type of contractual process. (TIF) [file pone.0035126.s008.tif]

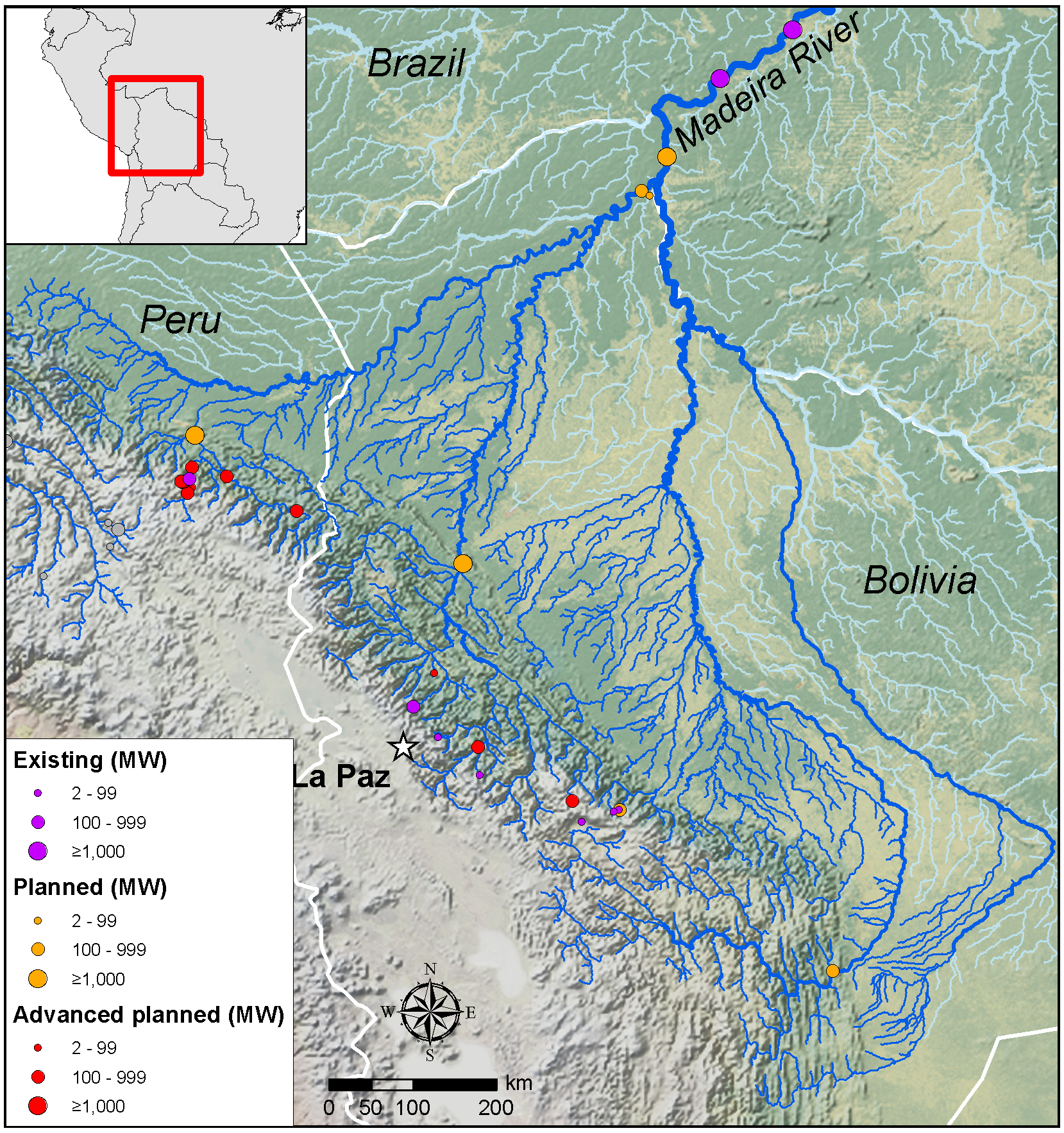

Supplement: Figure S9 — Hydroelectric dams of the Andean tributaries of the Madeira River Basin. Dams are grouped by status (Existing, Planned, and Advanced Planned) and size (2–99 MW, 100–999 MW, and ≥1,000 MW capacity). Advanced Planned corresponds to projects already under some type of contractual process. (TIF) [file pone.0035126.s009.tif]
