# Supplementary material for: Proliferation of Hydroelectric Dams in the Andean Amazon and Implications for Andes-Amazon Connectivity
Source: PLoS One. 2012 Apr 18;7(4):e35126. doi: 10.1371/journal.pone.0035126 (PMC3329437; doi:10.1371/journal.pone.0035126)
Supplement: Table S1 — List of all planned dams considered in the study and their key information and ecological impact scores. (DOC) [file pone.0035126.s010.doc]

**Table S1. List of all planned dams considered in the study and their key information and ecological impact scores.**

|  |  | **Size** |  |  |  | **Impact Analysis** | | | | | | |
| --- | --- | --- | --- | --- | --- | --- | --- | --- | --- | --- | --- | --- |
| **Country** | **Project** | **(MW)** | **Elev** | **Basin** | **Status** | **Fragment** | **AAC** | **Trans** | **Road** | **Enviro** | **Score** | **Indig** |
| Bolivia | El Yata | 6 | 116 | Mad | Planned | Mod | N | Y | N |  | **Mod** | N |
| Bolivia | Tahuamanu | 6 | 224 | Mad | Planned | Mod | N | Y | N |  | **Mod** | N |
| Bolivia | Pachalaca | 100 | 863 | Mad | Advance | High | N | Y | Y |  | **High** | N |
| Bolivia | San Jose | 126.9 | 1801 | Mad | Planned | Low | N | N | N |  | **Low** | N |
| Bolivia | Misicuni | 200 | 3696 | Mad | Advance | High | N | Y | Y | Y(PA) | **High** | Y |
| Bolivia | Miguillas | 250 | 1021 | Mad | Advance | Mod | N | Y | N |  | **Mod** | N |
| Bolivia | Rositas | 400 | 466 | Mad | Planned | High | N | N | Y |  | **Mod** | Y |
| Bolivia | Cachuela Esperanza | 990 | 111 | Mad | Planned | High | N | Y | N | Y (F & F) | **High** | N |
| Bolivia | Angosto del Bala | 1,600 | 176 | Mad | Planned | High | N | Y | Y | Y (PA) | **High** | Y |
| Bolivia | Rio Madera | 3,000 | 96 | Mad | Planned | Mod | N | Y | N | Y (F & F) | **Mod** | N |
| Colombia | Mitu | 2 | 175 | Vaup | Planned | Mod | N | Y | Y |  | **Mod** | N |
| Colombia | Andaqui | 687 | 323 | Caq | Planned | High | Y | Y | Y | Y (PA) | **High** | Y |
| Ecuador | Mayaicu | 2.27 | 1057 | Mara | Planned | Mod | N | Y | Y |  | **Mod** | Y |
| Ecuador | Nanguipa | 2.3 | 1214 | Mara | Planned | Mod | Y | Y | N |  | **Mod** | Y |
| Ecuador | Chorrillos | 3.96 | 1121 | Mara | Advance | Mod | Y | Y | N |  | **Mod** | N |
| Ecuador | Ambato | 4 | 3250 | Mara | Planned | Low | N | Y | N |  | **Low** | N |
| Ecuador | Sardinas | 4 | 1718 | Napo | Planned | Mod | Y | Y | N | Y (PA) | **Mod** | N |
| Ecuador | Huarhualla | 4.6 | 3009 | Mara | Planned | Low | N | N | N |  | **Low** | N |
| Ecuador | Collay | 5.8 | 2212 | Mara | Planned | Low | N | Y | N |  | **Low** | N |
| Ecuador | Tomebamba | 6 | 3022 | Mara | Planned | Low | N | Y | N |  | **Low** | N |
| Ecuador | Cebadas | 6.95 | 3159 | Mara | Planned | Low | N | Y | N |  | **Low** | N |
| Ecuador | Chinchipe | 8 | 1119 | Mara | Planned | Mod | Y | N | N |  | **Mod** | Y |
| Ecuador | Victoria-Quijos | 10 | 3702 | Napo | Advance | Mod | Y | Y | N | Y (PA) | **High** | N |
| Ecuador | Rio Verde Chico | 10 | 1590 | Mara | Advance | Low | Y | N | Y |  | **Mod** | N |
| Ecuador | Jondachi-Sardinas | 12.6 | 646 | Napo | Advance | Mod | Y | Y | N |  | **Mod** | Y |
| Ecuador | Chambo | 12.9 | 2932 | Mara | Planned | Mod | N | Y | N |  | **Mod** | N |
| Ecuador | Shincata | 14.3 | 2945 | Mara | Planned | Mod | N | Y | N |  | **Mod** | Y |
| Ecuador | Puela 2 | 14.8 | 2753 | Mara | Planned | Mod | N | Y | N |  | **Mod** | N |
| Ecuador | Bombuscara | 15 | 1281 | Mara | Planned | Mod | Y | Y | Y | Y (PA) | **High** | N |
| Ecuador | Palanda | 16.8 | 1424 | Mara | Advance | Mod | Y | Y | N |  | **Mod** | N |
| Ecuador | Jondachi-La Merced | 18 | 750 | Napo | Advance | Mod | Y | Y | Y |  | **High** | Y |
| Ecuador | Fatima | 20 | 912 | Mara | Advance | Mod | Y | Y | N |  | **Mod** | N |
| Ecuador | Mazar-Dudas-Llavircay | 20.9 | 2230 | Mara | Advance | Low | N | N | N |  | **Low** | N |
| Ecuador | Misahualli 2 | 21.1 | 895 | Napo | Planned | Mod | Y | Y | N |  | **Mod** | N |
| Ecuador | Topo | 22 | 1201 | Mara | Advance | Mod | Y | Y | Y |  | **High** | N |
| Ecuador | Valldolid | 22.3 | 1624 | Mara | Planned | Mod | N | Y | N |  | **Mod** | N |
| Ecuador | Due | 23.9 | 731 | Napo | Planned | Mod | Y | Y | Y | Y (PA) | **High** | Y |
| Ecuador | Chingual | 25.6 | 1509 | Napo | Planned | Mod | N | Y | N |  | **Mod** | N |
| Ecuador | Langoa | 26 | 2943 | Napo | Planned | Mod | Y | Y | Y | Y (PA) | **High** | Y |
| Ecuador | Cosanga | 27 | 1912 | Napo | Planned | Mod | Y | Y | N |  | **Mod** | N |
| Ecuador | Llanganates | 27.6 | 532 | Napo | Advance | Mod | N | Y | N |  | **Mod** | Y |
| Ecuador | Soldados Yanuncay-Minas | 27.8 | 2794 | Mara | Advance | Low | N | Y | N |  | **Low** | N |
| Ecuador | Sabanilla | 30.00 | 2767 | Mara | Advance | Mod | Y | Y | Y |  | **High** | N |
| Ecuador | Hidrogen | 31.4 | 2054 | Mara | Advance | Low | N | Y | Y | Y (PA) | **High** | N |
| Ecuador | Sucua | 31.6 | 780 | Mara | Planned | Mod | Y | N | N |  | **Mod** | Y |
| Ecuador | Numbala | 39.2 | 2050 | Mara | Planned | Mod | Y | Y | Y |  | **High** | N |
| Ecuador | La Barquilla | 40.1 | 866 | Napo | Planned | Mod | Y | Y | N |  | **Mod** | Y |
| Ecuador | Cuyes | 50.6 | 1097 | Mara | Planned | Mod | Y | Y | N |  | **Mod** | Y |
| Ecuador | Isimanchi | 51.1 | 1473 | Mara | Planned | Mod | Y | Y | N |  | **Mod** | N |
| Ecuador | Sizaplaya | 52.1 | 2081 | Napo | Planned | Mod | Y | Y | N |  | **Mod** | N |
| Ecuador | Sonaderos | 70.8 | 1045 | Mara | Planned | Mod | Y | Y | N |  | **Mod** | N |
| Ecuador | Jatunyacu | 74.1 | 554 | Napo | Planned | Mod | Y | Y | Y |  | **High** | Y |
| Ecuador | Las Cidras | 77.3 | 1206 | Mara | Planned | Mod | Y | Y | Y |  | **High** | N |
| Ecuador | Negro | 97.7 | 926 | Mara | Planned | Mod | Y | Y | N |  | **Mod** | N |
| Ecuador | Quijos-Baeza | 100 | 1236 | Napo | Advance | High | Y | Y | Y |  | **High** | N |
| Ecuador | Delsitanisagua | 115 | 831 | Mara | Advance | High | Y | N | N |  | **Mod** | N |
| Ecuador | Lligua-Muyo | 170 | 1823 | Mara | Planned | Low | N | N | N |  | **Low** | N |
| Ecuador | El Retorno | 261 | 1603 | Mara | Planned | Mod | Y | N | N |  | **Mod** | Y |
| Ecuador | Cedroyacu | 270 | 1935 | Napo | Planned | High | N | Y | Y |  | **High** | Y |
| Ecuador | Cardenillo | 340.9 | 1045 | Mara | Advance | Low | N | N | N |  | **Low** | N |
| Ecuador | Sopladora | 487 | 1101 | Mara | Advance | Low | N | N | N |  | **Low** | N |
| Ecuador | Gualaquiza | 661 | 679 | Mara | Planned | High | Y | Y | N |  | **High** | N |
| Ecuador | San Miguel | 686 | 607 | Mara | Planned | High | Y | Y | N |  | **High** | N |
| Ecuador | Catachi | 748 | 995 | Napo | Planned | High | Y | Y | Y | Y (PA) | **High** | Y |
| Ecuador | San Antonio | 760 | 523 | Mara | Planned | High | Y | Y | N |  | **High** | Y |
| Ecuador | Zamora Salto 2 | 917 | 526 | Mara | Planned | High | Y | Y | N |  | **High** | Y |
| Ecuador | Zamora Salto 1 | 924 | 688 | Mara | Planned | High | Y | Y | N |  | **High** | N |
| Ecuador | Zamora Salto 3 | 1,015 | 385 | Mara | Planned | High | Y | Y | N |  | **High** | Y |
| Ecuador | Zamora San Juan Bosco | 1,028 | 749 | Mara | Planned | High | Y | Y | N |  | **High** | Y |
| Ecuador | Naiza | 1,039 | 326 | Mara | Planned | High | Y | Y | N |  | **High** | Y |
| Ecuador | Verdeyacu Chico | 1,173 | 1039 | Napo | Planned | High | Y | Y | Y |  | **High** | Y |
| Ecuador | Coca Codo Sinclair | 1,500 | 1268 | Napo | Advance | High | Y | Y | Y |  | **High** | N |
| Peru | Santa Catalina | 4 | 694 | Ucay | Planned | Mod | Y | Y | Y | Y (PA) | **High** | N |
| Peru | Colpa | 4.8 | 2282 | Mara | Advance | Mod | N | N | N |  | **Low** | N |
| Peru | Naranjos II | 5.8 | 1271 | Mara | Advance | Mod | Y | Y | N |  | **Mod** | N |
| Peru | Huasahuasi I | 7.86 | 2545 | Ucay | Advance | Low | N | N | N |  | **Low** | N |
| Peru | Huasahuasi II | 8 | 2396 | Ucay | Advance | Low | N | N | N |  | **Low** | N |
| Peru | Pias | 12.6 | 1584 | Mara | Advance | Mod | Y | Y | Y |  | **High** | N |
| Peru | Carcapata III | 12.8 | 1973 | Ucay | Advance | Low | N | N | N |  | **Low** | N |
| Peru | Las Orquideas 1 | 13.8 | 949 | Mara | Planned | Mod | Y | Y | Y |  | **High** | N |
| Peru | Pias II | 16.6 | 1298 | Mara | Advance | Mod | Y | Y | N |  | **Mod** | N |
| Peru | Tulpac/Palenque (Pusac) | 20 | 921 | Mara | Advance | Mod | Y | Y | N |  | **Mod** | N |
| Peru | Ayna | 20 | 1308 | Ucay | Advance | Mod | N | N | N |  | **Low** | N |
| Peru | Angel I | 20 | 2532 | Mad | Advance | Low | N | Y | Y |  | **Mod** | N |
| Peru | Angel II | 20 | 2240 | Mad | Advance | Low | N | Y | Y |  | **Mod** | N |
| Peru | Angel III | 20 | 1952 | Mad | Advance | Low | N | N | N |  | **Low** | N |
| Peru | Renovandes H1 | 20 | 1009 | Ucay | Advance | Mod | Y | Y | N |  | **Mod** | N |
| Peru | Centauro I y III | 25 | 2963 | Mara | Advance | Mod | Y | N | N |  | **Mod** | N |
| Peru | Santa Fortunata | 25.2 | 2818 | Mara | Planned | Mod | N | N | N |  | **Low** | N |
| Peru | Uchuhuerta | 30 | 2542 | Ucay | Planned | Low | N | Y | N |  | **Low** | N |
| Peru | Las Joyas | 61 | 1307 | Mara | Planned | Mod | Y | Y | Y |  | **High** | N |
| Peru | La Virgen | 64 | 1037 | Ucay | Advance | Low | N | N | N |  | **Low** | N |
| Peru | Lavasen Quishuar | 64.2 | 1278 | Mara | Planned | Mod | Y | Y | Y |  | **High** | N |
| Peru | Pucara II | 69.9 | 3589 | Ucay | Planned | Mod | N | Y | N |  | **Mod** | N |
| Peru | Pumayacu Cachiyacu | 80 | 203 | Mara | Planned | Mod | N | Y | Y |  | **Mod** | Y |
| Peru | Santa Teresa | 90.7 | 1313 | Ucay | Advance | Low | N | N | N |  | **Low** | N |
| Peru | Marañon | 96 | 2851 | Mara | Advance | Mod | Y | N | N |  | **Mod** | N |
| Peru | Pacobamba | 98.7 | 1285 | Ucay | Planned | Mod | Y | Y | N |  | **Mod** | N |
| Peru | Oreja De Perro 1 | 100 | 991 | Ucay | Advance | High | Y | Y | Y |  | **High** | N |
| Peru | El Caño | 100 | 1518 | Ucay | Planned | High | N | N | N |  | **Low** | Y |
| Peru | Mayo II | 110 | 440 | Mara | Advance | High | Y | Y | Y |  | **High** | Y |
| Peru | Mayo I | 120 | 786 | Mara | Advance | High | Y | N | N |  | **Low** | N |
| Peru | San Gaban IV | 130 | 2589 | Mad | Advance | Low | N | N | N |  | **Low** | N |
| Peru | Puchca | 140 | 2622 | Mara | Planned | High | Y | Y | N |  | **High** | N |
| Peru | San Gaban 1 | 150 | 2096 | Mad | Advance | Low | N | N | N |  | **Low** | N |
| Peru | Pucara (Salcca) | 150 | 3926 | Ucay | Advance | Mod | N | N | N |  | **Low** | N |
| Peru | Mazan | 150 | 88 | Napo | Planned | Low | N | Y | Y |  | **Mod** | N |
| Peru | Yanamayo | 160 | 1885 | Mara | Planned | High | Y | Y | N |  | **High** | N |
| Peru | Curibamba | 163 | 1329 | Ucay | Advance | Low | N | Y | N |  | **Low** | N |
| Peru | Belo Horizonte | 180 | 693 | Mara | Advance | High | N | Y | N |  | **Mod** | N |
| Peru | San Gaban III | 187.9 | 740 | Mad | Advance | Low | N | N | N |  | **Low** | N |
| Peru | Retamal | 188.6 | 2526 | Ucay | Planned | Low | N | N | Y |  | **Low** | N |
| Peru | Llata 2 | 200 | 2501 | Mara | Planned | High | Y | Y | N |  | **High** | N |
| Peru | Llata 1 | 210 | 2787 | Mara | Planned | High | Y | N | N |  | **Mod** | N |
| Peru | Pulpería | 220 | 2054 | Mara | Planned | High | Y | Y | Y |  | **High** | N |
| Peru | Patas 2 | 240 | 1114 | Mara | Planned | High | Y | Y | N |  | **High** | N |
| Peru | Chusgón | 240 | 1268 | Mara | Planned | High | Y | Y | Y |  | **High** | N |
| Peru | Man (Mantaro) 270 | 286 | 1057 | Ucay | Planned | Mod | N | Y | N |  | **Mod** | N |
| Peru | Bolívar | 290 | 993 | Mara | Planned | High | Y | Y | N |  | **High** | N |
| Peru | Pauya-Cushabatay | 300 | 162 | Ucay | Planned | High | Y | Y | Y |  | **High** | N |
| Peru | Rupac | 300 | 1698 | Mara | Planned | High | Y | Y | N |  | **High** | N |
| Peru | Sandia Ina 30-Ina 40 | 315 | 1136 | Mad | Advance | High | N | Y | N |  | **Mod** | N |
| Peru | Patas 1 | 320 | 1309 | Mara | Planned | High | Y | Y | N |  | **High** | N |
| Peru | Illapani | 327.9 | 867 | Ucay | Advance | High | Y | Y | N |  | **High** | Y |
| Peru | Yangas | 330 | 968 | Mara | Planned | High | Y | Y | Y |  | **High** | N |
| Peru | Santa Rosa | 340 | 966 | Mara | Planned | High | Y | Y | Y |  | **High** | N |
| Peru | Oreja de Perro | 350 | 1308 | Ucay | Advance | High | Y | Y | Y |  | **High** | N |
| Peru | Pión | 350 | 585 | Mara | Planned | High | Y | Y | N |  | **High** | N |
| Peru | Chaglla | 360 | 863 | Mara | Advance | High | N | Y | Y |  | **High** | N |
| Peru | Ina 65-88-90 | 380 | 631 | Mad | Advance | High | N | Y | Y |  | **High** | N |
| Peru | San Pablo | 390 | 1575 | Mara | Planned | High | Y | Y | N |  | **High** | N |
| Peru | Cerro El Aguila | 402 | 1336 | Ucay | Advance | Mod | N | Y | N |  | **Mod** | N |
| Peru | Tambo 60 | 579 | 207 | Ucay | Planned | High | Y | Y | N | Y(Flood) | **High** | Y |
| Peru | Del Norte | 600 | 610 | Mara | Advance | High | Y | Y | Y |  | **High** | N |
| Peru | Chadin 2 | 600 | 661 | Mara | Advance | High | Y | Y | Y |  | **High** | N |
| Peru | Tambo - Puerto Prado | 629 | 305 | Ucay | Planned | High | Y | Y | N | Y(Flood) | **High** | Y |
| Peru | Veracruz | 730 | 502 | Mara | Advance | High | Y | Y | N |  | **High** | N |
| Peru | Pongo de Aguirre | 750 | 152 | Mara | Planned | High | Y | Y | Y |  | **High** | N |
| Peru | Vizcatán | 750 | 608 | Ucay | Planned | Mod | N | Y | Y |  | **Mod** | N |
| Peru | Santa Maria | 750 | 1535 | Ucay | Advance | High | Y | Y | Y |  | **High** | N |
| Peru | Cuquipampa | 800 | 686 | Ucay | Planned | Mod | N | Y | Y |  | **Mod** | N |
| Peru | Cumba 4 | 825 | 484 | Mara | Advance | High | Y | Y | N |  | **High** | N |
| Peru | La Balsa (Balsas) | 915 | 829 | Mara | Planned | High | Y | Y | N |  | **High** | N |
| Peru | Sumabeni | 1,074 | 445 | Ucay | Planned | High | Y | Y | N |  | **High** | Y |
| Peru | Tam40 | 1,286 | 295 | Ucay | Advance | High | Y | Y | Y | Y(Flood) | **High** | Y |
| Peru | Paquitzapango | 1,379 | 404 | Ucay | Planned | High | Y | Y | Y | Y(Flood) | **High** | Y |
| Peru | Inambari | 1,500 | 384 | Mad | Planned | High | N | N | Y | Y(Flood) | **High** | Y |
| Peru | Rentema | 1,525 | 333 | Mara | Planned | High | Y | Y | N | Y(Fish) | **High** | Y |
| Peru | Mainique (Urubamba) | 1,548 | 659 | Ucay | Planned | High | Y | Y | N |  | **High** | Y |
| Peru | Escuprebraga | 1,800 | 245 | Mara | Planned | High | Y | Y | Y | Y(Fish) | **High** | Y |
| Peru | Manseriche | 4,500 | 157 | Mara | Planned | High | Y | Y | Y | Y(Fish) | **High** | Y |

Data sorted by Country and then Size. Columns from left to right: country, project name, size (MW), elevation (meters), river basin (Ucayali, Marañon, Napo, Caqueta, and Madeira tributaries), status (Advance and Planned), and multi-factor impact analysis scores. For status, Advance corresponds to advanced stage projects under some type of contractual process, while Planned refers to projects with initial designs but not yet under any type of contract. Impact analysis categories contributing to final score refer to 1) fragmentation index, 2) Andes-Amazon connectivity, 3) transmission line access, 4) road access, and 5) significant known environmental issue (Protected Area, flooding, migratory fish). See Methods for scoring details. The last column refers to the titled indigenous territory analysis (see Methods for details), which did not contribute to the final score.
